# Exploring the usage of learning resources by medical students in the basic science stage and their effect on academic performance

**DOI:** 10.1186/s12909-024-05511-1

**Published:** 2024-05-15

**Authors:** Sabin Kumar Ranabhat, Mohan Lal Kunjukrishnan, Muskan Dubey, Vernon Curran, Arun Kumar Dubey, Neelam Dwivedi

**Affiliations:** 1 Department of Pathology, Xavier University School of Medicine, Oranjestad, Aruba; 2 Department of Anatomy, Xavier University School of Medicine, Oranjestad, Aruba; 3 Xavier University School of Medicine, Oranjestad, Aruba; 4 Department of Medical Education, Memorial University of Newfoundland, Newfoundland, Canada; 5 Department of Pharmacology, Xavier University School of Medicine, Oranjestad, Aruba; 6 Department of Medicine, OSCE and SP Program, Xavier University School of Medicine, Oranjestad, Aruba

**Keywords:** USMLE Step 1, Learning resources, Textbooks, Review books, Medical students

## Abstract

**Background:**

The United States Medical Licensing Examination (USMLE) step 1 is one of the two examinations written after completion of the first two years (basic science stage) of medical school to be eligible to apply for residency training in the USA. A huge number and types of study materials are available to prepare for the exam which might confuse students choosing a resource.

We investigated learning resources being used by the third and fifth-semester medical students and their association with academic performance. We also compared learning resources and exam scores of high-performing and low-performing students.

**Methods:**

Data collection was done using structured (quantitative study) and semi-structured (qualitative study) questionnaires during a face-to-face interview. This article is about the quantitative part which was designed as a correlational study. Single factor one-way analysis of variance (ANOVA), Pearson correlation coefficient test, T-test, and Fisher’s exact test were used to analyze the data.

**Results:**

About half of all students used three or more commercial resources dealing with the same content. A weak negative correlation was observed between the number of commercial resources and the exam scores, especially when the number of these resources was three or more (*r* = -0.26). The mean exam score of textbook users was statistically significantly higher than the mean score of textbook non-users (*p* = 0.01). The usage of textbooks was statistically significantly higher in the cohort of top performers in comparison to the rest of the students (*p* = 0.006). In addition to less usage of textbooks, the mean number of review books was higher in the group of weakest students (2.84 versus 3.7; *p* = 0.75).

**Conclusions:**

Most students did not use professional textbooks and about half used too many commercial review resources. While the former fact was significantly associated with poor academic performance, the later fact had weak negative correlation with exam score.

Pedagogical interventions are urgently needed to make the right type of learning resources available by making professional textbooks more USMLE-oriented and helping the students choose the best and right number of resources for optimum academic performance. By fulfilling the observed needs of the students in this way, they might feel empowered because of self-determination which will motivate studies.

**Supplementary Information:**

The online version contains supplementary material available at 10.1186/s12909-024-05511-1.

## Introduction

The United States Medical Licensing Examination (USMLE) is written by the United States as well as international medical graduates at the end of the two-year basic science stage of medical school to enroll in three to seven more years of medical training (residency) in different branches of medical science in the USA. In 2022, that number stood at 53,881, which is much higher than the residency slots available which is 39,205 [1]. The successful residency match rate in 2022 for US medical graduates, US International Medical Graduates (US IMG), and non-US IMGs was 92.9%, 61.4%, and 58.1% respectively [2]. However, there is a silver lining in the cloud: according to a report from the Association of American Medical Colleges in 2017, there will be a shortage of up to 104,900 physicians in the USA by 2030 [3].

To maintain the competitive edge, a student needs to score higher than the others in Step 2 Clinical Knowledge (CK) even though USMLE Step 1 score reporting has been changed to a simple pass-fail from the previous three-digit numeric scoring on January 26, 2022 [4]. Passing step 1 in the first attempt is also not an easy job which is necessary to make oneself competitive in the residency match.

Numerous commercial organizations and individuals have developed a multitude of resources that are supposed to help students with their studies and preparation for the USMLE examinations. These resources include review books, question banks, and audio–video resources based on the fact that more than 70% of students are multimodal learners as described by the VARK model: Visual, Auditory, Reading and Writing, and Kinesthetic. These resources give students the flexibility to choose one according to their needs [5–7]. However, an important caveat is these resources are written concisely mainly to help students revise the already learned USMLE content in a short period (e.g., a few weeks) before USMLE step 1 and as supplementary resources to traditional textbooks during the basic science years [8]. Nevertheless, many medical students are using these resources as the primary learning platforms from an early period of the first year itself or in the second year well before the dedicated USMLE study period. Such a phenomenon is called “Step 1 climate” [9].

Several factors contribute to this phenomenon as explained hence onward. The influence of the USMLE test preparation industry and social media often promote a humanistic approach to learning, emphasizing student choice and control over resources. This resonates with students who desire the freedom to select materials they find suitable. Many concise review resources prioritize testable, high-yield information, presented in formats like bullet points and buzzwords. While seemingly efficient, this approach can have drawbacks. By focusing solely on “high-yield” material, students might miss out on broader foundational knowledge and the context required to truly understand tested concepts. These resources may not cover all testable topics, and low-yield information, although not explicitly tested, can be crucial for comprehending the bigger picture. While these concise resources might seem convenient, they may not equip students with the comprehensive understanding needed to excel in their studies and future careers. The result is a potential gap in students’ knowledge base due to lack of in-depth study [9–11]. Unlike review resources designed for rapid revision and recall, textbooks support in-depth exploration of a topic by delving into the historical context and theoretical foundations of each topic. They provide multifaceted explanations and diverse perspectives. Through this comprehensive approach, textbooks empower students to transcend rote memorization and cultivate a deep, nuanced understanding of the subject matter [12].

Jeyaraju et al. analyzed 201 studies on USMLE exams and found that the most important factor for success was a good foundational base of knowledge supported by regular and continuous habits of learning over time from comprehensive resources. Practice tests were also found to be helpful as the number of practice test items and practice exam scores positively correlated with higher USMLE scores [12]. Other researchers have also found that the foundation of knowledge developed through an accredited curriculum during the basic science years is the most important factor for success in USMLE steps [8, 13, 14]. Nevertheless, it does not mean commercial review resources have no place in medical education. Some researchers have demonstrated that the usage of such resources along with traditional learning material helps students score higher marks in USMLE exams [15, 16]. Using question banks, which is called retrieval practice, has been shown to improve long-term retention and recall of previously learned information which can be explained by Cognitivist learning theory [14, 17, 18].

The National Board of Medical Examiners (NBME) and the Federation of State Medical Boards (FSMB), the organizations that provide oversight of the USMLE examinations, have changed the scoring system of Step 1 to a pass-and-fail from a 3-digit-scoring system to encourage students to pay more attention to the accredited school curriculum and discourage too much focus on Step 1 score and unwise usage of commercial test resources [19]. However, due to the following reasons, this might not be enough to bring students to the classroom environment from step 1 climate. The fact that principles and facts of basic sciences are the core basis of clinical sciences learning and thus success with the USMLE step 2, knowledge acquired during the first two years at medical school is vital to score better in the step 2 exam. This reason and the increment in the passing score of Step 1 from 194 to 196 and a similar change in Step 2 CK score from 209 to 214 will ensure the ever-increasing proliferation of commercial test preparation resources tailor-made for USMLE step 1 [20]. Program directors from now on will shift their focus to Step 2 score for the screening of potential candidates for interviews to screen thousands of candidates for a handful of residency positions [21, 22]. Before the change in scoring system, program directors used step 1 score as a first-line screening tool that could compare applicants objectively [23, 24].

Therefore, it would be better to maintain the status quo on the numeric scoring system of USMLE and find other ways to strike a balance between traditional teaching–learning methods and the usage of commercial test resources by students.

### Statement of problem/rationale of the study

As educators guiding students through the foundational basic sciences in our medical school, we have witnessed firsthand the pervasive influence of the “step 1 climate.”

Students have become increasingly drawn to the perceived efficiency of these concise review resources. They often express frustration with the time commitment required to grasp complex concepts from textbooks. This preference for quick solutions has manifested in several concerning ways: reduced class attendance, textbook neglect, and utilization of too many concise resources.

Our research aims to dissect this problem from multiple angles. We want to determine if there’s a tipping point where relying on too many concise resources becomes counterproductive for learning and by analyzing how high-performing and low-performing students utilize learning resources, we want to identify potential strategies that contribute to academic success. Armed with objective research findings, we will be able to engage in data-driven discussions with students, faculty, and the USMLE preparation industry to create a learning environment that fosters a strong foundation in basic sciences while adequately preparing students for the USMLE.

While the current literature acknowledges student confusion regarding resource selection, a comprehensive investigation into the potential downsides of relying heavily on commercial review resources and neglecting textbooks is lacking.

This research has the potential to significantly impact medical education by providing much-needed clarity on the role of commercial review resources, textbooks, and Question Banks, and their impact on student learning outcomes. Additionally, by comparing resource utilization patterns between student groups, we can glean valuable insights into effective learning strategies.

### The purpose of the study was

- Explore the types of learning resources medical students are using in the basic science stage of medical school and examine their effect on academic performance.

### The specific objectives of the research were

1. Explore what learning resources students use.
2. Examine whether the types of resources utilized influence exam scores.
3. Compare learning resources and exam scores of high-performing students and low-performing students.

## Methodology

### Research design and variables

Research design: It was a mixed quantitative and qualitative study. For the quantitative part, a correlational study design was used to find out the association between independent and dependent variables (Table 1). This research paper is about the quantitative part. The qualitative part of the study will be submitted to a journal for publication in the second stage.

**Table 1:** Research variables

Category of variable	Independent (predictor) variable	Dependent (outcome) variable
1. Textbooks	Usage of textbooks: yes and no	Exam score
2. Commercial review resources	up to two, up to three, and up to and above four	Exam score
3. Question banks	up to two, one and more, two and more	Exam score
4. Age-groups	< 24 years, 25 to 34 years, ≥ 35 years	Exam score
5. Gender	Male or female	Exam score

### Study site and ethical approval

The study was conducted at Xavier University School of Medicine, Aruba after ethical approval was received from the Institutional Review Board (IRB) of Xavier University School of Medicine (Study ID number: XUSOM/IRB/2023/03/001). The University offers a four-year undergraduate medical program, Doctor of Medicine (MD). Students complete the first two years, the basic science stage, in Aruba. At the time of this research, students were studying in the first semester, the third semester, and the fifth semester.

### Study participants

Twenty-nine (*n* = 29) out of 30 Doctor of Medicine students in the 3rd semester and fifteen students (*n* = 15) out of 19 in the 5th semester were included in the study.

The sample size was calculated with the below-mentioned formula with following parameters: confidence level (1-alpha) 95%, margin of error 5%, and population size 49.

The participation was voluntary. Participants were selected randomly by a simple random sampling technique using a table of random numbers. Written consent was taken before data collection. Participants were anonymized by giving each of them a unique code as described below: the first participant of the third semester was given the code “M3.1” and the first participant of the fifth semester was given the code “M5.1”. Subsequent participants were coded serially.

Top-performing students were identified as those who scored 65% or more in the most recent NBME exam. Those students who scored less than 35% were identified as the weakest students. The score thresholds were selected for the purpose arbitrarily.

### Study resources

In this research, three types of resources have been studied: professional USMLE-oriented textbooks, commercial review resources, and question banks. Learning resources have been categorized as mentioned below based on a pilot study, relevant literature, and personal experience (Table 2).

**Table 2:** Types of learning resources students are using in the basic science stage

Category of learning resources	Learning resources
1. USMLE-oriented textbooks	a). Paperback or digital format: - Rapid Review of Pathology by Dr Edward Goljan, - Lippinkot Pharmacology, - Lippinkot Biochemistry, - Keith and Moore Anatomy, - Review of Medical Microbiology and Immunology by Levinson b). Online format: Amboss, USMLE-Rx
2). Commercial review resources	- First Aid for the USMLE Step 1, - Boards and Beyond review, - Ninja Nerd, - Med School Bootcamp, - BRS books, Sketchy, - Anky cards, Pathoma, - Dirty Medicine, and Kaplan books
3). Question banks	- UWorld, - USMLE-Rx, - Amboss

### Exam scores

NBME nervous system exam scores were taken as the reference for MD 3 semester students and NBME cardiovascular system exam scores were taken as the reference for MD 5 semester students in order to compare with the usage of learning resources.

### Data collection

Data collection was undertaken using a questionnaire survey completed during a face-to-face interview by the lead author adhering to the interview protocol. Questionnaire items included questions regarding demographic variables, types of learning resources students were using, study behavior, and perceptions about the different types of learning resources, lectures, and exams.

A pilot study was conducted among 6 students to validate the questionnaire to check the internal consistency and reliability of survey questions. The pilot study was also used to assess question clarity, answer options, and the overall flow of the survey. These students were not included in the main study. Some questions were deleted, some were modified, and some were added to the questionnaire after gaining new insights from the data of the pilot study. The questionnaire also underwent expert review to enhance its content validity and ensure it appropriately captures the target construct.

Responses to the qualitative part of the study were audio-recorded.

### Survey questions

There are in total 23 items in the questionnaire. Two items are open-ended questions intended to gain insight into students’ perceptions about learning resources and study behavior (qualitative study). The rest 21 items are devised to collect quantitative data. Thirteen of these 21 items are related to learning resources which is the main subject matter of the current manuscript. The questionnaire has been uploaded as a supplementary file.

### Data analysis

Quantitative data were analyzed by descriptive methods: frequencies, percentages, mean, and significance tests where applicable.

Single factor one-way analysis of variance (ANOVA) was used to determine whether there was a statistically significant difference between mean exam scores among different age groups. The Pearson correlation coefficient test was completed to analyze the correlation between the number of review books used and the exam score.

An independent samples T-test was used to compare the mean score between the two groups of students with the following variables viz., review books, Q Banks, textbooks, and gender. F test was used before the T-test to find out whether the variance of the two populations was equal or not.

Fisher’s exact test was used for two pairs of nominal data when the sample size was not large enough to use the Chi-square test. An alpha level of 0.05 was used for all statistical tests of significance. The qualitative part of the study will be presented in another article.

## Results

### Demographic profile of participants

A total of 44 medical students of the basic science stage were enrolled in the study. The largest number of participants were in the age group of 25 to 34 years. Age-related mean, median, and mode values are 27.18, 26.5, and 26 respectively. Table 3 contains the summary of demographic information of participants.

**Table 3:** Demographic characteristics of participants

Variable	Number (*n* = 44)	Percent
**Age (completed in years)**		
< 24	13	29.6
25–34	26	59.0
35 and above	05	11.4
**Gender**		
Male	20	45.4
Female	24	54.6
**Semester**		
MD3	29	65.9
MD5	15	34.1

Table 4 consists of information about age-groups and mean score for each age-group, mean exam score for males and female students and score categories. *P* value was calculated for different categories wherever applicable.

**Table 4:** Exam scores of participants

**Total Students**	**Range of exam scores**	**Mean exam score**	**Statistical test**
44	26 to 86	54.4	
**Age-groups**	**Mean exam score**	**Number of students**	^*^P value = 0.52 (*F* value 0.66; F crit vale = 3.22)
< 24	58.2	13 (29.5%)	
25–34	51.9	26 (59.1%)	
35 and above	54.0	05 (11.4%)	
**Gender**			^**^Two tail *P* value = 0.52
Male (*n* = 20)	52.35		
Female (*n* = 24)	55.45		
**Score categories**	**Number of students**	**Mean exam score**	^*^P-value = 0.005 (*F* value: 18.5; F crit: 5.9)
a). > 65	13 (29.5%)	73.6	
b). 50 to 64	10 (22.7%)	57.9	
c). 35 to 49	15 (34.1%)	43.8	
d). < 35	06 (13.6%)	30.8	

^*^ANOVA-Single factor was used for calculating the *P* value

^**^Independent samples T-test assuming unequal variances was used for calculating the *P* value

**Fig. 1:**
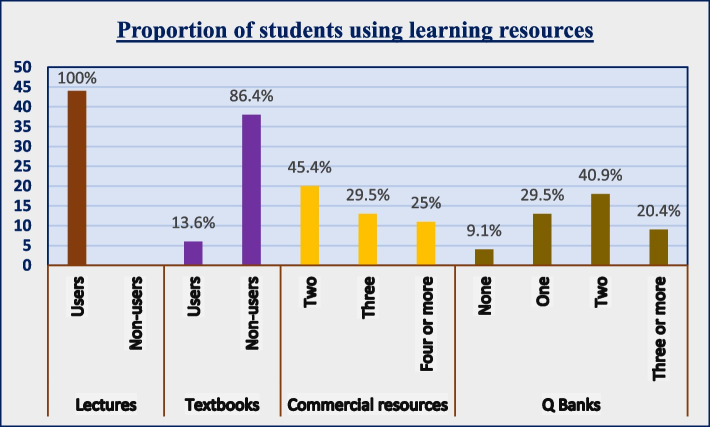
Clustered column chart- proportion of students using learning resources

Figure 1 shows the major types of learning resources and percentage of students using them.

In Table 5, the Pearson correlation coefficient was calculated for categories under ‘commercial review resources’. The mean exam score was calculated for three groups of students who differed by the number of review books they were using. A similar statistical analysis was done with question banks. The findings are shown in the table.

**Table 5:** Exam score and related variables (*n* = 44)

Variable	Mean Score	Number (%)	Statistical test
**Commercial review resources**			Pearson correlation coefficient
a). Up to two	NA	20 (45.4%)	*r* = 0.12
b). Up to three	NA	33 (75%)	*r* = -0.26
c). Up to and above four	NA	44 (100%)	*r* = -0.23
**Commercial review resources**			^*^P value = 0.2 (*F* value 1.64; F crit vale = 3.22)
a). One or two books	58.45	20 (45.4%)	
b). Three books	48.5	13 (29.5%)	
c) Four or more books	52.5	11 (25%)	
**Q Banks**			Pearson correlation coefficient
a). Up to two	NA		*r* = -0.19
a). One and more	NA		*r* = 0.19
b). Two or more	NA		*r* = 0.45
**Q Banks**			^*^P value = 0.48 (*F* value 0.83; F crit vale = 2.83)
a). None	58.5	04 (9.1%)	
b). One Q Bank	50.9	13 (29.5%)	
c). Two Q Banks	52.1	18 (40.9%)	
d). Three and four	60.4	09 (20.4%)	
**Textbooks (users vs non-users)**			^**^Two tail *P* value = 0.01
a). Users	72.6	06 (13.7)	
b). Non-users	51.1	38 (86.3)	

In this study population, there was no effect of age on the score obtained in the exams

^*^ANOVA-Single factor was used for calculating the *P* value

^**^Independent samples T-test assuming unequal variances was used for calculating the *P* value

### Usage of commercial review resources (third-party resources): Table 5

All students used commercial review resources. Some students used a greater number of these resources than others. The use of many review resources was found to be associated with decreased exam scores as tested by the Pearson correlation coefficient (*r* = -0.26). The mean score of students who used one or two review books was higher than the mean score of students who used more than three review books although the difference was not statistically significant when tested by ANOVA-single factor test.

The most commonly used review book was First Aid (88.6%) followed by Boards and Beyond (56.8%) and Ninja Nerd (27.3%). Other less frequently used review resources were Med School Bootcamp, BRS books, Sketchy, Anky cards, Pathoma, Dirty Medicine, and Kaplan books.

**Fig. 2:**
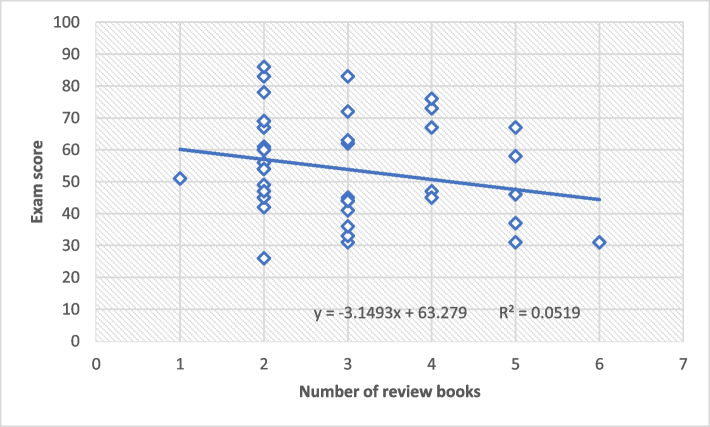
Scatter plot- correlation between the number of review books (x-axis) and exam score (y-axis)

Figure 2 shows that as the number of review books increase, exam score of students decreases.

### Usage of questions banks: Table 5

Approximately ninety percent of 90% of students used question banks. The number of different Q Banks ranged from one to four. The mean number of Q Banks among users was 1.7. The most used Q Bank was UWorld (45.4%), the second was USMLE Rx (31.8%) and the third was Amboss (25%). The mean score of students who used three or four Q Banks (60.4) was higher than the mean score of students who used two or fewer (52.1) although the difference was not statistically significant when tested by ANOVA-single factor test (*P* value: 0.48). There was a weak negative correlation between the usage of two or fewer Q Banks and the exam score (*r* = -0.19). However, there was a weak positive correlation between the usage of three or more Q Banks and the exam score which was not statistically significant.

### Usage of textbooks: Table 5

Only six students (13.6%) said they used three or more textbooks daily as the main learning resource, while the rest did not use textbooks as the main learning resource. Independent samples t-test assuming unequal variance was used to analyze the statistical significance between the mean scores of these two populations. The mean exam score of textbook users was found to be statistically significantly higher than the mean score of textbook non-users: 72.6 versus 51.1 (*P* value = 0.01).

### Usage of lecture notes

All students used lecture notes for internal examinations.

### Top-performing students versus the rest of the students (Table 6)

**Table 6:** Comparison of top-performing students with the rest

**Top performers vs the rest**	**Exam score criteria**		**Mean exam score **
a). Top performers (*n* = 13)	≥ 65		73.6
b). Rest of the students (*n* = 31)	< 65		45.8
	**Usage of textbooks**		* **Two-tailed ** ***P*** ** value**
	Yes	No	0.006
Top performers (*n* = 13)	5 (38.46%)	8 (61.54%)	
Rest of the students (*n* = 31)	1 (3.22%)	30 (96.78%)	
**Analysis of textbook usage within top performers’ cohort**	**Usage of textbooks among top performers**		
	Yes	No	
	5	8	
	Mean exam score among top performers		0.13
	Textbook users	Non-users	
	78	70.8	
	**Usage of First Aid review book**		0.6
	Yes	No	
Top performers (*n* = 13)	12	1	
Rest of the students (*n* = 31)	26	5	
	**Usage of Question Bank book**		1.0
	Yes	No	
Top performers (*n* = 13)	12	1	
Rest of the students (*n* = 31)	28	3	
**Q Banks**	**The mean number of Q Banks**		0.28
a). Top performers (*n* = 13)	2.07		
b). The other cohort (*n* = 31)	1.65		
**Study hours: regular days**	**Mean study hours: regular days**		0.53
a). Top performers (*n* = 13)	3.3		
b). Rest of the students (*n* = 31)	3.7		
**Study hours during exams**	**Mean study hours during exams**		0.71
a). Top performers (*n* = 13)	7.5		
b). The other cohort (*n* = 31)	7.8		
**Review books**	**The mean number of review books**		0.75
a). Top performers (*n* = 13)	2.84		
b). The other cohort (*n* = 31)	2.96		
c). Score < 35 cohort (*n* = 6)	3.7		

^*^Statistical tests that were used for data in this table:

1. Fisher’s Exact test was used to calculate the *P* value for the data related to textbooks, First Aid review books, and Question Bank books

2. Independent samples T-test assuming unequal variances was used to calculate the *P* value for the data related to the means of two groups

3. Independent samples T-test assuming equal variances was used to calculate the *P* value for the data related to two means of review books

There were 13 students in the cohort of top performers and 31 in the rest of the cohort.

The usage of textbooks was statistically significantly higher in the cohort of top performers in comparison to the rest of the students (*P* value: 0.006): Table 6.

### Usage of first aid review book: Table 6

Twelve out of thirteen top-performing students used first aid while twenty-six out of thirty-one in the other cohort used this review book. The difference was not statistically significant as calculated by Fisher’s exact test (*P* value = 0.6).

### Usage of Q Bank: Table 6

Twelve out of thirteen top-performing students and twenty-eight out of thirty-one in the other cohort used at least one Question Bank book. The difference was not statistically significant as calculated by Fisher’s exact test. The difference in mean number of Q Bank books between the two groups was also not statistically significant (*P* value = 0.28).

There was no statistically significant difference between the means of the two groups regarding study hours and the number of review books (P values 0.71 and 0.75).

## Discussion

### Varied learning resources: what do students choose?

With growing mobile device usage and greater internet connectivity, there has been a radical change in the type and nature of learning resources used by medical students. In the past, students had to rely solely on didactic lectures and print books for learning purposes, however, this is simply not the case anymore. Students have a wide array of choices ranging from professional textbooks in digital format and paperback to concise commercial resources in paperback, audio, and video format, and question bank books. The practice of blended learning where students use both traditional and new digital learning tools has been well-established universally [25–28]. How has this change affected academic performance concerning the type and number of resources utilized? This is the question that has prompted this research.

### The impact of commercial review resources usage on academic performance

It has been found that most students choose concise commercial resources over comprehensive professional textbooks as the source to acquire knowledge. In the current study, only 13.6% of students used professional textbooks along with commercial resources which reflected similar findings from a study (15%) in a German medical school [29]. Makus D et al. from the University of Ottawa reported students spend 63% of their time on commercial resources [30]. Snow CE et al. and Scott K et al. found that students are less motivated to use textbooks when commercial review resources are available and are perceived as more effective [27, 31]. These findings are very important from an academic viewpoint because the mean score of the group of students who used professional textbooks along with other commercial review resources was statistically significantly higher than the mean score of textbook nonusers in this research.

Students are more likely to choose too many commercial review resources of the same scope due to several reasons: availability of numerous brands of commercial resources, digital advertisements, and exchange of information on social media and peer effect [6]. In this study, 54.5% of students used three or more commercial resources dealing with the same content. This trend might be causing harm to the students because a negative correlation, although weak, was observed between the number of commercial resources and the exam scores, especially when the number of these resources was three or more. The mean exam score for this group of students who used three or more of these resources was found to be lower than the mean exam score for students who used only one or two of these resources. However, the difference in mean scores between the two groups was not statistically significant. This suggests that the usage of a greater number of commercial review resources may not offer any advantage in academic success. Ikonne U et al. found a statistically significant positive correlation between usage of review resources and academic performance in the case of both first- and second-year medical students. However, the correlation was not studied in relation to the number of resources [16]. Bauzon, J et al. found that higher exam scores were moderately associated with less utilization of commercial review resources [32].

### The impact of question bank usage on academic performance

Medical schools and licensing board examinations including USMLE in developed nations and some developing countries utilize multiple choice questions in the form of clinical vignettes in all types of theoretical examinations. Students use many types of question bank resources to prepare for these types of exams. Using question banks has been shown to improve long-term retention and recall of previously learned information [15]. However, we found no statistically significant difference in the mean exam score among several cohorts concerning the use of question banks: non-users, students using one Q bank, two Q banks, and three or more Q banks. We cannot explain the reason for this dissimilar finding in our study.

Because 61.3% of students were using more than two or more Q banks, it was not possible to study the effect of a particular type on the exam score. Multiple regression analysis completed at the University of Alabama School of Medicine found that exposure of students to USMLE-type questions throughout the preclinical stage resulted in improved academic performance [32]. In a study that involved two groups of students writing the Emergency Medical Services fellowship exam, the group that completed the prescribed question bank obtained an overall 12% higher pass rate than the group of students who did not. However, it is not clear from the study whether the same subject experts who created the question bank were also involved in formulating questions for the fellowship exam. The most important fact is these students used question banks along with regular learning resources [33].

### High-performing students versus rest of the students

Our study has a total of 44 participants who had exam scores from 26 to 86. We arbitrarily separated the top 13 students who had exam scores more than 65 and analyzed different variables head-to-head with the rest of the students who had scores less than 65. The difference in means of exam scores of these two groups is statistically significant and the only factor which has a significant effect on the exam scores is the usage of textbooks. Approximately 38% of students in the top-performing cohort used textbooks in comparison to just 3.2% in the cohort of the rest of the students. This same variable was also examined among students within the top-performing cohort. Textbook users had higher mean exam scores, although the difference is not statistically significant. We did not observe differences concerning other variables (usage of Q Banks, review books, and study hours) between the top-performing cohort and the rest of the students.

### High-performing students versus low-performing students

Similarly, the top-performing cohort was compared with the cohort of bottom-level students who scored less than 35 on the exam. The observation regarding textbook usage was the same as described above. In addition, the mean number of review books was higher in the bottom-level students than in the top-performing cohort although the difference was not statistically significant.

### Usage of textbooks and their impact on academic performance

In a study done at the University of Michigan, Rafel JB et al. found that 77% of students who used commercial step 1 resources including concise review books and question banks early during the basic science stage along with preclinical curriculum, achieved higher step 1 scores [15]. In our study group, 13.7% of students used professional textbooks along with concise review books and question banks, and they had a significantly higher mean exam score. Parry S et al. have a slightly different finding than that of Rafel JB et al. In their study carried out in a Midwestern Medical School, the researchers found that preclinical grade was strongly associated with USMLE step 1 validating the usage of textbooks as the most important factor for success and interestingly, question bank usage but not the usage of review resources had a statistically significant positive impact on the scores [14].

According to the self-determination theory (SDT) proposed by Deci and Ryan, students feel motivated to learn when three psychological needs are fulfilled: autonomy to choose learning resources (feeling of self-governed), feeling competent to perform learning tasks, and the ability to engage with peers and teachers [34]. Professors at The University of Sydney School of Medicine opine that pedagogy should be aligned with self-determination theory and teachers should help students choose commercial resources of high quality to make learning more effective [25].

### Embracing ‘step 1 climate change’ and ways to mitigate it

Step 1 climate is pervasive across all US and Canadian medical schools, and many parts of the world. Ikonne U et al. from Eastern Virginia Medical School found that students started using review resources from the first year. The authors recommend designing learning sessions and curricula to accommodate review resources to make students effective self-regulated learners [16]. Makus D et al. from the University of Ottawa suggest restructuring curriculum design and delivery in order to make room for nontraditional learning resources in the official curriculum [30]. Rather than denying and ignoring the “step 1 climate change”, it’s a good idea to embrace it and try to make it better.

In 2016, educators from the University of North Carolina School of Medicine undertook an audit of the basic science stage to evaluate content discrepancies between their curriculum and “high-yield” Step 1 topics. A highly popular review book among students “First Aid for USMLE Step 1 “and USMLE Step 1 score reports of students were used for this purpose. They also made customized exams administered by NBME, the same organization that oversees USMLE, mandatory to students. Many students-centered pedagogical interventions were also made: training students on test-taking skills, providing a subscription to USMLE-oriented resources namely USMLE-Rx for content and UWorld for multiple choice questions, and one-to-one counseling focused on the needs of a particular student. Because of the restructuring of the curriculum in line with USMLE step 1, the school achieved a resounding success with a first-time pass rate of 99.4% in 2018 which was 95% in 2016, and a numeric score of three points more than the national average [35].

## The uniqueness of this study

The current study delves into the usage of learning resources by medical students in the basic science stage and its impact on their academic performance from multiple angles. While many studies have been done on this topic, the authors of this survey did not find any article in the literature regarding the comparison of high-performing students and low-performing students. Observations have been documented in the results section and described in the discussion section. These observations can help teachers and students make adjustments to enhance teaching–learning activities.

Apart from that, few studies have analyzed data regarding the number of commercial review resources and their effect on academic performance. We have divided students into four groups based on the number of review resources they are using, the data has been analyzed in detail.

## Limitations of this study

a We might get a clearer picture if research is done in many medical schools across all continents so that the findings can be generalized for all medical students in the basic science stage.
b Mean exam score was found higher in the cohort of high-performing students than in the cohort of low-performing students because of the usage of textbooks in the former. However, to eliminate the confounding bias due to dissimilar levels of ‘intelligence’ among participants, this conclusion should be validated by a case–control study with a large sample size taking care to match the entry-time ‘Grade Point Average (GPA)’ of the participants in the two cohorts.

## Conclusion

In this study, medical students in the basic science stage have provided themselves with the autonomy of choosing learning resources in line with Humanistic learning theory to fulfill unique needs demanded by the USMLE step 1 examination. However, it has been observed that the majority of students did not use professional medical textbooks and about half of the students used three or more commercial review resources. Both facts were significantly associated with poor academic performance.

It is therefore vitally necessary that medical schools, educators, and authors take the following pedagogical interventions to make the right type of learning resources available and help the students choose the best resources for optimum academic performance in the basic science stage of the MD program. In this way, students might feel empowered because of self-determination and stay on course because of the behaviorist paradigm from the subject experts.

1. Medical school curriculum should be restructured to incorporate balanced usage of commercial review books and NBME-styled multiple-choice questions apart from professional textbooks to fill the void of observed needs and felt needs of medical students.
2. Students should be discouraged from using too many parallel commercial review resources.
3. To draw students away from commercial review resources, authors should write textbooks in such a way that they are structured, aligned with the content of USMLE step 1, and concise without losing the depth and breadth of knowledge.

### Supplementary Information

Supplementary Material 1.

## Data Availability

All the data underlying the results are available which can be obtained through the corresponding author.
