# Supplementary material for: Exploring the usage of learning resources by medical students in the basic science stage and their effect on academic performance
Source: BMC Med Educ. 2024 May 15;24:543. doi: 10.1186/s12909-024-05511-1 (PMC11097491; doi:10.1186/s12909-024-05511-1)
Supplement: Supplementary file 1 — Supplementary Material 1. [file 12909_2024_5511_MOESM1_ESM.docx]

**Appendix: Questionnaire**

**A]. Interview protocol**

| 1. | Write the date, place, name of the interviewer, and code number of the interviewee.  M3.1.S1= first participant in stratum 1 of MD3 semester;  M5.1.S1 first participant of stratum 1 of MD4 semester.  Other participants are coded consecutively. |
| --- | --- |
| 2. | Introduce the interviewee to the aim and objectives of the study |
| 3. | Take the consent of the interviewee for an interview and record the interview. Assure the participant of anonymity. |
| 4. | **The standard procedure to be followed by the interviewer:**  a. Ask one or two ice-breaker questions to build rapport: How is everything going? How is your studies going on?  b. Listen attentively. Do not get distracted or bored.  c. Use active listening techniques: nodding your head, smiling.  d. Do not answer questions from the interviewee in relation to the ‘outcome’ part of the research.  e. Do not rush the interview session.  f. Do not ask more than one question at a time.  g. Do not ask for opinions on behalf of the group they are a part of.  h. Ask probing questions (follow-up questions) where needed to elicit more detailed responses.  i. Close the interview by thanking the participant. |
| 5. | Audio record the interview.  If a participant does not give consent for audio record, interview will be recorded by hand. |

**B].** **Semi-structured guide for qualitative data**

| **A]. Usage of learning resources:**  - Your opinion about different learning resources:  - Books for each subject: textbooks, review books, Q banks.  - Lecture notes of subjects: how useful are they?  - Internal exams; NBME exams. Clinically Correlated Presentation (CCP): |
| --- |
| **B]. Study behaviour:** |

**C]. Structured questionnaire for quantitative data**

| **1. The number of subjects lecture notes are aligned to the NBME exam and thus are useful for:**   1. Lecture notes of only one subject are useful. 2. Lecture notes of only two subjects are useful. 3. Lecture notes of only three subjects are useful. 4. Lecture notes of only four subjects are useful. 5. Lecture notes of only five subjects are useful. 6. Lecture notes of only six subjects are useful. 7. Lecture notes of all subjects are useful.   **2. How many hours do you study per weekday** **when there is no upcoming exam?**   1. <2 b. 2-5 c. >5   **3. On which learning resource do you spend your time during regular days: proportion of time on**  **each resource in percentage?**  a. Lecture notes: d. Textbooks:  b. First aid: e. Qbank:  c. Other review books:  **4. How many hours do you study per weekday during exam time?**   1. <5 hours b. 5 to 8 hours c. >8 hours   **5. On which learning resource do you spend your time during internal exams time: proportion of**  **time on each resource in percentage?**  a. Lecture notes: d. Textbooks:  b. First aid: e. Qbank:  c. Other review books:  **6. On which learning resource do you spend your time during NBME exams time: proportion of time**  **on each resource in percentage?**  a. Lecture notes: d. Textbooks:  b. First aid: e. Qbank:  c. Other review books:  **7. How many hours do you study per weekend day when there is no upcoming exam?**  a. <2 b. 2 – 5 c. >5  **8. Do you use question bank?**   1. Yes b. no   **9. If yes, which question bank do you use?**  Answer:  **10. Why do you use Q banks?**   1. To prepare for USMLE step I b. To prepare for NBME exams 2. Because seniors tell me to study Q bank d. Because friends use them. 3. Because teachers tell me to use them. f. To test my knowledge 4. To improve my test taking skill   **11. How do you use Q bank?**   1. after learning content from textbook b. before learning content from textbook   c. sometimes before and other times after learning the content. d. I don’t use it at all  **12. Do you use review books?** a. yes b. no  **13. If yes, which review book/s do you use?**  Answer:  **14. If yes, why do you use review books?**   1. It takes a shorter time to study. Textbooks are bulky. 2. Friends use them. c. Seniors tell me to study these books. 3. To prepare for USMLE step I, d. To prepare for NBME exams. 4. Lectures do not cover all the topics. Not to miss any topic that might be asked in the NBME exam. e. Because teacher/s tell me to study these books. |
| --- |
| **15. How many subjects you do not want to miss a class of?**   1. One b. Two c. Three d. Four e. Five f. Six g. seven   **16. What do you do during the semester break?**  a. Study b. enjoy c. Research d. Observership in a hospital e. all  **17. How important is a good grade in exams?**  “Not at all important,” “Slightly Important,” “Important,” “Fairly Important,” and “Very Important,” |
| **18. What is the objective of basic sciences?**  a. to pass USMLE step I b. to build foundation for clinical sciences. c. both d. no idea  **19. How many subjects the internal exams are aligned with NBME exams of?**  a. one subject b. two subjects c. three subjects d. four subjects e. five subjects f. six subjects g. seven  **20. What is the name of the text book/s for each subject?**   1. For pathology: 2. For physiology: 3. For biochemistry: 4. For anatomy: 5. For pharmacology: 6. For microbiology: 7. For ICMPD:   **21.What are the barriers to study?**  Answer: |

**Demographic questions:**

Age: Sex: Semester:

Date:

**Lead author: Dr Sabin Kumar Ranabhat; XUSOM.**
